# Supplementary material for: Are carbon emissions trading and green financial instruments synergistic? -Comprehensive quantitative research based on content analysis
Source: PLoS One. 2024 Mar 7;19(3):e0298601. doi: 10.1371/journal.pone.0298601 (PMC10919601; doi:10.1371/journal.pone.0298601)
Supplement: S1 Data — (ZIP) [file pone.0298601.s001.zip › Data/地级市Z_F工作报告.zip!/╣π╬≈/╖└│╟╕█╩╨/└┤╫╘╣1⁄2╓┌║┼í╛┬φ┐╦╩2╛▌═°í┐_2014.docx]

各位代表：

现在，我代表市人民政府向大会作工作报告，请予审议，并请市政协委员和列席会议的同志提出意见。

2013年工作回顾

过去的一年，面对复杂多变的国内外经济形势，我们在自治区党委、政府和市委的坚强领导下，坚持以科学发展观为统领，按照“稳中求进”的总基调，以迎接建市20周年、建港45周年为契机，全面推进各项工作，经济社会发展呈现稳中加快的良好态势，较好地完成市五届人大四次会议确定的各项任务。

——经济实现逆势上扬。全市地区生产总值突破500亿元大关，达525亿元，增长12.4%；工业总产值突破1000亿元大关，增长23.3%；财政收入59.3亿元，增长13.1%，总量全区排名又上升一位；全社会固定资产投资475亿元，增长14.5%；港口货物吞吐量1.06亿吨，增长5%；地区生产总值、规模以上工业总产值、外贸进出口总额等11项重要指标总量或增幅位居全区前列。

——干好干成一批大事。以“双二十”大事为抓手，带动全局发展。三大天字号工程实现突破，钢铁冷轧项目开工建设、核电项目全面进入设备安装阶段、金川40万吨铜冶炼项目投产。一批百亿元项目建设加快，金源一期、盛隆技改一期顺利竣工，信润石化、海森特海工装备、科元新材料、澳加粮油等项目开工建设。南防高铁开通运营，我市步入高铁时代；防东高速建成通车，实现高速公路县县通，全市形成1小时经济圈。

——工业实力显著增强。以培育14个百亿元企业为重点，全力扶持企业提质增效。超100亿元企业增加1家，达3家；超10亿元企业增加2家，达12家；超亿元企业增加12家，达106家；全市规上工业企业达162家。企业效益持续向好，全市规上工业经济效益综合指数达681.3%，连续7年排全区第一。

——民生福祉不断提升。民生投入保持快速增长，达到63亿元，增长20%。居民收入水平继续快速提升，城镇居民人均可支配收入增长10%；农民人均纯收入增长13.5%，连续6年保持全区第一。民生保障范围继续扩大，享受低保比例全区第一，基本社保覆盖98%，新农合参合率99%，居全区前列。保障水平持续提高，低保发放标准全区第二。实施281个民生项目，10件为民办实事项目如期完成。

一年来，我们主要做了以下工作：

（一）强力抓好项目建设。完善项目工作机制，统筹推进项目建设，解决征地拆迁等瓶颈问题。全年下达“双二十”项目788项，新开工285项，续建305项，竣工231项。全市在建项目投资总额超过5000亿元，投资额5000万以上的在建项目223项，投资总规模为建市以来最高，这批项目为我市长远发展奠定了坚实的基础。

（二）全力推动开放合作。全面加快试验区建设，先行先试取得新突破。个人人民币跨境结算、进境种苗（景观树）指定口岸和边境旅游异地办证业务获批准实施。13个国家部委或自治区厅局同意制定专门支持政策。12项重点规划编制工作稳步推进，试验区发展规划等4项规划编制完成。东兴-芒街跨境经济合作区建设稳步推进，建设方案已报国家审批。一批标志性项目加快建设，国际旅游集散中心等项目竣工，北仑河国际商贸城、红木文化产业园等重点项目加快推进。加大与东盟交流，密切与港澳台、中南、西南等周边地区合作，成功承办中国-东南亚民间高端对话会，举办第七届中国-东盟社会发展与减贫论坛、国际海上龙舟节、中越青年大联欢等活动，得到各方高度评价。

（三）着力完善城市功能。围绕“三岛三湾一核心六组团”的城市布局，完善城市配套，丰富城市内涵。城市建成区面积达51平方公里，城镇化率达54%，获“广西园林城市”荣誉称号。加快推进一批城建重点项目，开工400项，竣工110项。伏波文化公园、龙马明珠公园等4大主题公园基本建成，科技馆、文化艺术中心等7大场馆建成开放。抓好市政设施建设，市区供水、排污、燃气、通信等一批管网工程加快推进。实施“十大城建惠民工程”，改造47条小街小巷，新建20多项便民设施。

（四）大力发展第三产业。加大政策支持力度，全力促进第三产业增量提质。商贸业稳步发展，全年社会消费品零售总额81.4亿元，增长14.2%。实现外贸进出口总额43亿美元。边境贸易进出口总额241.5亿元，增长19.6%。西湾集贸市场、中心区集贸市场等19个专业市场推进顺利。旅游业取得新突破，接待游客965万人次，增长19.6%。开办外国人落地签证，复航防城港至下龙湾游轮。江山半岛旅游度假区黄金周日接待游客超过10万人，被评为“中国十佳海洋旅游目的地”。积极实施“引金入港”工程，全市新增6家小额贷款公司，金融组织体系日益完善。房地产稳步发展，完成投资100亿元，完成销售面积146万平方米。文化创意、设计评估、中介服务等其他服务业加快发展。

（五）做大做强港口物流。实施“以港立市”战略，加快建设现代化枢纽大港。加强港航基础设施建设，401-407#泊位、云约江南作业区1#泊位工程竣工，20-22#煤炭专业码头改造工程完成。企沙东岛、企沙南港区前期工作稳步推进。物流业加快发展，东湾物流加工区、公车物流园、冲仑物流园开发建设加快。铁路货场扩建工程、北部湾大货车物流服务中心竣工运营，东湾仓储物流基地、北部湾现代物流中心等重点项目加快推进。全市物流企业达350多家。

（六）全面夯实三农基础。农林牧渔业总产值111.6亿元，增长5.6%。粮食生产保持稳定。加快农业产业化，实现“红金系列”种植规模150万亩，糖料蔗种植63万亩，水产养殖28万亩，发展林下种养121万亩。扶持农业龙头企业37家，6家企业被认定为自治区级重点龙头企业。狠抓农民增收工程，建设“万元片区”20多万亩、“万元村屯”300个、畜牧养殖“万元片区（场）”860多个。海水养殖稳步发展。农村基础设施建设加强，除险加固病险水库35座，完成农村人饮工程144个，解决4万人安全饮水问题。全市行政村公路水泥硬化率达98%。新建新农村示范点15个。扎实开展扶贫工作，全市新增脱贫人口1.4万。

（七）深入开展美丽家园活动。投入资金3.6亿元，实施“美丽乡村”、“美丽企业”、“美丽海洋”等“八大美丽工程”，城乡环境显著改善。派出322个工作组688名工作队员进村入户。出动20多万人次，清理垃圾近14万吨。加强城市综合整治，成立市城市综合执法局，拆除违法建筑约21万平方米。加大西湾海域环境整治力度，完成清理面积2.1万亩。

（八）统筹发展社会事业。科技创新取得新成就，新增6个工程技术研究中心培育点，明华科技孵化器投入使用并通过自治区级认定，实现科技企业孵化器零的突破。发明专利申请量、每万人发明专利拥有量增速居全区前列。教育事业稳步发展，全市新建5所学校，市四中、市特殊教育学校基本建成，市五中、六中等学校开工建设。卫生事业取得新进步，医药卫生体制改革顺利推进，在全区率先发行“金穗新农合一卡通”。“幸福家园”等人口计生工作扎实推进。文化建设有新突破，民族题材音乐剧《过桥风吹》、歌舞剧《疍家秀》首演，音乐短剧《香糯香》获全国“群星奖”优秀奖。全民健身活动蓬勃开展，水营体育馆、珠河社区体育馆主体工程基本完成。全市城镇新增就业2.5万人，城镇居民失业率1.76%，就业率全区最高。农村劳动力新增转移就业1.8万人。第三次开展城镇职工免费健康体检。保障房建设开工率达到100%，棚户区改造工作得到国务院督查组肯定。强化环保倒逼机制，抓好环境安全隐患大排查大整改，全面完成污染减排任务，加强安全生产工作，全市没有发生重大安全生产事故和环境安全事故。成功抗击“海燕”等8次强台风袭击。加大食品药品监管治理力度，深化食品药品监管体制改革，提高食品药品安全保障水平。深入开展矛盾纠纷的排查化解工作，上思县“一村一警”工作成为自治区推广样板。严厉打击各种刑事犯罪活动，加强反走私综合治理，强化边境管控，保持边疆稳固安宁。

（九）切实加强政府建设。认真落实中央“八项规定”精神，工作作风进一步转变。提高政府审批效能，组织召开全市项目联合审批会9次，市本级45个审批单位和530项审批事项全部进入政务服务中心办理。县（市、区）向乡镇下放审批权限33项。加快推动政务服务向基层延伸，全市80%以上的乡镇（街道）、30%的行政村（社区）建成政务服务中心。举办全市首届“政务公开日”活动。新成立十万山瑶族乡，防城镇撤镇设立水营、文昌、珠河3个街道。江平盐场改制工作完成。强化财税管理，提高理财水平，有力支持社会事业和经济建设发展。积极化解财政风险，偿还政府性债务13.5亿元。厉行节约，“三公”经费保持零增长。严格编制管理，严控财政供养人数。停止新建楼堂馆所，开展办公用房清理工作，腾退办公用房1万多平方米。自觉接受人大和政协的监督，人大代表建议和政协委员提案办结率100%。

一年来，外事、侨务、统计、审计、国资、司法、工商、质监、海关、检验检疫、海事、通信、邮政、供销、地震、气象、人防、消防、保密、决策咨询、机关事务管理、社会科学、新闻出版、地方志、档案、民族宗教、残疾人、妇女儿童等工作取得新进步。国防教育和后备力量建设深入开展，边海防基础设施建设进一步加强，双拥共建工作取得新成绩。

各位代表！过去一年，我市在艰难形势下经济保持平稳较快发展，社会各项事业不断进步，成绩来之不易。这是自治区党委、政府和市委正确领导的结果，是市人大、市政协有效监督、大力支持的结果，是中直、区直单位和驻军驻警支持帮助的结果，是各级各部门和全市人民团结拼搏、真抓实干的结果。在此，我谨代表市人民政府，向全市广大干部群众，向解放军指战员、武警官兵和公安民警，向各民主党派、工商联、无党派人士、人民团体，向所有关心、支持、参与我市现代化建设的各界人士和海内外朋友，表示衷心的感谢和崇高的敬意！

在肯定成绩的同时，我们也清醒地看到，我市经济社会发展仍然面临较多困难和问题。主要是：大部分产业项目还处在投入阶段，税收贡献不足，投资可持续增长的压力越来越大；产业集聚效应不明显；土地、资金、人才等要素制约比较突出；节能减排压力加大；城市功能配套不完善，社会民生事业发展滞后；一些部门和个别干部执行力不强，“四风”问题在不同程度存在。对此，我们要千方百计加以克服和改进，努力把政府工作做得更好，绝不辜负全市人民的期望！

2014年工作安排

2014年是贯彻落实党的十八届三中全会精神的开局之年，也是我市全面建成小康社会“三步走”路线图迈入“第二步”的第一年。随着国家全面深化改革扩大开放、中国-东盟合作进入新的“钻石十年”、国家建设21世纪“海上丝绸之路”和广西北部湾经济区、东兴试验区加快建设，广西打造我国中南、西南地区新的战略支点步伐加快，我市发展又迎来大有可为的战略机遇期，我们必须增强机遇意识、忧患意识，团结拼搏，开拓进取，努力开创发展新局面。

今年政府工作的总体要求是：深入贯彻落实党的十八大、十八届三中全会和自治区党委十届四次全会、人大十二届三次会议，以及市委五届五次全会的精神，以科学发展观为统领，坚持“稳中求进、改革创新”的总基调，围绕“两个建成”目标，按照“改革开放求突破、转型升级上台阶”的要求，着力提速度、优结构、增效益、惠民生，为加快迈入沿海开放城市第一集团奠定坚实基础。

今年我市的经济社会发展主要预期目标为：地区生产总值600亿元，增长12%；财政收入65.8亿元，增长11%；全社会固定资产投资537亿元，增长13%；全部工业总产值1257亿元，增长25.5%；社会消费品零售总额92.4亿元，增长13.5%；外贸进出口总额51.6亿美元，增长20%；实际利用外资3.2亿美元，增长10%；港口货物吞吐量1.15亿吨，增长8.9%；城镇居民人均可支配收入27110元，增长11%；农民人均纯收入9580元，增长12%；居民消费价格涨幅控制在5%以下；城镇登记失业率控制在4%以内；人口自然增长率控制在10‰以内；万元地区生产总值能耗和主要污染物排放量控制在自治区要求范围内，各项社会事业取得新成效。

为此，我们将突出抓好以下九项重点工作：

一、以龙头企业为引领，加快构筑临海工业体系

进一步实施和拓展“226工程”，引进和壮大一批龙头企业，延伸产业链，加快临港工业集聚，打造现代版临海工业基地。

壮大龙头企业。以钢铁、有色金属两大千亿元产业为引领，加快形成千百亿元产业集群。抓好钢铁基地建设，推进200万吨冷轧项目建设，开工建设500万吨高炉项目，加快配套码头、厂区道路等设施建设；支持金川项目发展壮大，尽快开工二期项目，加快铁路、供电、路网、配套码头建设，金川项目年内产值要超百亿元；推进红沙核电500KV输出线路、应急道路和核辐射监测站建设，争取开工核电二期项目；基本建成信润石化项目；启动盛隆技改二期项目；加快海森特海工装备、科元新材料、中海油LNG储运项目的建设。通过龙头产业项目的牵引，壮大产业规模，确保今年产值超百亿元企业达4家，超10亿元企业达14家，超亿元企业达110家，规上工业企业达180家。

抓好产业园区建设。以企沙工业区、大西南临港工业区两大千亿元园区为重点，加快理顺园区管理体制，完善园区产业规划，加强水、电、路、排污等基础设施配套，增强园区承载力。企沙工业区要围绕钢铁、有色金属等重大产业，开工建设盛弘机械制造、冷轧包装、钢材配送、武钢建工防城港联合钢结构项目。推进中核华原硫酸循环利用项目。争取开工中电二期等一批项目，同时引进一批上下游项目，延长产业链，加快形成千亿元园区。大西南临港工业区要抓好与东湾物流加工区的合并工作，以冶金、磷化、石化、粮油、物流等产业为重点，开工建设金源不锈钢项目，推进澳加粮油、枫叶粮油二期、中外运东湾仓储物流基地等项目建设，加快磷化产业园整体搬迁。争取年内实现工业总产值750亿元，2年内成为我市首个千亿元产业园区。冲仑、公车物流园要完善物流服务设施，引进一批大型物流企业，推进东盟农产品国际采购物流园、汇圣啤酒厂等重点项目建设。茅岭工业园要加快开工建设标准化厂房、矿业物流园等项目。上思县工业集中区要依托自身优势，重点发展资源深加工产业，做大做强蔗糖、水泥建材、林板林化三大产业，抓好朗姆酒、华威木业等项目建设。规划建设高新技术产业园、深圳（防城港）产业园。

加快产业转型升级。发挥钢铁、有色金属等重大项目带动作用强的优势，延伸产业链，引进更多关联企业，提升产业发展层次。大力发展循环工业，做好节能减排，推动企沙工业区循环经济产业园、华润（防城港）循环经济产业园建设。加快建设金川配套固废、菌海铜渣综合利用项目，努力把金川、盛隆冶金、昌菱制糖公司等企业建设成自治区循环经济示范企业。

推进非公经济加速发展。抓紧出台非公有制经济发展支持政策，完善领导干部联系非公企业制度。为非公经济创造更加宽松的发展环境，在降低准入门槛、规范市场秩序等方面下功夫，千方百计解决好融资难、用地难、用工难、办事难、税费重“四难一重”问题。实施“抓大壮小扶微”工程，加大政策扶持和服务力度，设立产业基金，千方百计吸引一批国内外大型民营企业到我市投资发展。

二、实施“十百千项目计划”，促进投资拉动增长

以十大龙头项目为引领，以百项重大项目为重点，全力推进千项规模以上项目建设，全面上项目促投资，掀起新一轮项目建设热潮，确保年度完成投资537亿元。

培育十项龙头项目。突出抓好钢铁基地、红沙核电、金川有色金属、盛隆冶金、金源镍业、信润石化、科元新材料、海森特海工装备、港务集团、中海油LNG储运等10项产值百亿元以上的龙头产业项目建设，继续引进和培育一批龙头项目，力争年内龙头产业项目达到13项以上。

落实百项重点项目。围绕工业产业、港口发展和城市建设等方面，谋划落实百项投资额3亿元以上的重点项目。推进针鱼岭大桥、月亮岛主题公园、华美立家建材市场等36个续建项目，开工中宝煤化工、江山旅游接待基地等59个项目；抓好企沙东岛现代化工基地、防城港30万吨码头等12个项目的前期工作。

实施千项规上项目。加快推进千项投资500万元以上的项目建设。抓好239个产业项目，完成投资262亿元；抓好319个城建项目，完成投资123亿元；抓好123个文化旅游项目，完成投资20亿元；抓好114个民生项目，完成投资14亿元；抓好57个港口项目，完成投资29亿元。加强项目储备，提高招商水平，引进更多更好的项目落户我市。

强化项目建设保障。完善工作机制，落实项目联系制度和集中审批制度，提高项目前期工作质量；加强土地收储，推进连片征地开发。坚持安置优先，解决好被征地群众的就业、社保等问题，启动北港安置区四期工程建设，规划建设江山半岛、公车物流园安置新区。抓好低丘缓坡荒滩等未利用地开发利用试点，破解项目用地指标难题。做大做强市级融资公司，年内确保三公司一中心总资产达到450亿元以上。强化重点项目融资，争取融资100亿元以上。

三、加快试验区建设，提高开发开放水平

发挥试验区政策优势和品牌效应，解放思想，敢闯敢干，不断拓展先行先试领域，努力在扩大开放合作上实现新突破。

着力提升规划策划水平。加快编制空间规划及产业规划，完善江平新城、金滩国际旅游岛等一批规划。抓好北投海产品加工园区的前期工作。实质性推进红木产业园、东盟汽贸城、富安居家居中心、国际商贸城等一批重点项目。精心策划组织各种专场推介会，加大宣传力度，打响试验区品牌，争取把试验区建成我国沿边地区资金、人才、项目聚集地。

全面推进先行先试。抓好自治区34条支持政策的落实，努力把政策优势转化为发展优势。加快推进工商登记制度改革、外国人口岸签证等一批先行先试政策。抓住国家建设沿边金融综合改革试验区的机遇，推动跨境人民币结算、人民币与越南盾兑换特许等方面的改革创新。做好水果、粮食等产品进口指定口岸申报工作。努力增加互市贸易的品种和额度，扩大边境贸易规模。争取国家和自治区在政策、资金、项目等方面给予更多支持。

努力搭建开放合作平台。以“两区两园”为引领，推动一批开放合作平台的建设。抓好东兴-芒街跨境经济合作区申报工作，推进北仑河二桥和口岸联检设施建设，争取成为国家第一批跨境经济合作区。启动综合保税区建设前期工作。尽快做好深圳（防城港）产业园选址，开展前期工作。开工建设冲榄工业园基础设施。加快规划建设企沙东岛。抓好中国-印尼经贸合作区的策划工作。加快口岸开放升格，争取防城港口岸扩大开放范围方案获国家批准，抓好垌中口岸升格为国际口岸的申报，推动里火边境合作区建设，恢复开通滩散互市区。

积极拓展对外交流合作。加大与欧美、中东、东亚以及港澳台、珠三角、中南西南等国家和地区的交流合作。举办好中国-东盟边境商贸·旅游博览会、金滩论坛等活动。深化与东盟各国的全方位交流合作，主动参与和融入21世纪“海上丝绸之路”建设。面向东盟，服务中南、西南，大力发展进出口加工、商贸服务、现代物流、跨境旅游和金融业等产业。

四、彰显海湾特色，打造宜居城市

依托“三岛三湾”自然优势，挖掘海洋文化内涵，高标准规划、高质量建设、高水平管理，打造靓丽的海湾城市。

突出海湾特色。结合山海相依、河海相连的地形地貌，完善城市总体规划，精心编制一批城市详细规划、专项规划。规划建设要充分体现山海元素和特点，做到显山露水、凸显边海色调风格。强化西湾整治，完善景观布局，把北部湾海洋文化公园、仙人山公园、龙马明珠公园、伏波文化园及西湾城市沙滩等打造成环西湾景观带。推进绿色慢道等休闲空间建设，打造百里黄金海岸。利用历史人文资源，推进海上胡志明小道、汉唐文化古迹等项目建设。全力推进创建“国家园林城市”工作，加快沙潭江湿地公园、金花茶公园等“五个一批”绿化项目建设。

完善城市基础设施。强化城区交通建设，西湾大道、东湾大道、文昌大道等道路年内竣工，推进防城至江山一级公路、江山半岛环岛东路改扩建工程建设，加快常山新区路网、城南新区路网建设，实施一批城区小街小巷道路改造。加强城市中心区功能配套，完善市场、学校、医院等设施，营造良好宜居环境。加快污水管网改造，建成大西南污水处理厂、江平污水处理厂，新建企沙污水处理厂、江山污水处理厂。加快城市电力管网配套和通讯光缆入地管道建设。发展公共交通，规范公交车和出租车的运营行为，方便市民出行。

推进新型城镇化。规划建设沿海、沿江、沿边城镇带，加快产城融合，壮大城镇产业。抓好企沙、江平、那良、扶隆、茅岭、在妙等中心镇（乡）建设，培育一批经济强镇、特色名镇。健全乡镇规划建设管理办法，完善城乡基础设施，规范农村建房，引导农民保护耕地、节约使用土地。统筹城乡发展，引导农民进城务工，推动农村富余劳动力向城镇转移。东兴要加快风貌改造，在城市色调、建筑立面中融入东盟元素，体现民族边关风情，把东兴建设成现代版的国际门户城市，打造成广西的看点和亮点。上思要注重生态保护与挖掘民族内涵，建设生态养生县城和民族风情小镇。加快港口区步行街、桃花湾片区等精品街区建设，推进防城区老城区改造升级。

五、振兴第三产业，增强经济发展活力

把第三产业作为优化经济结构的战略重点，出台第三产业发展振兴计划，制定优惠政策，促进商贸、旅游等产业大发展。

提升旅游业。全力建设“两地三线四区”旅游精品工程，建设跨境旅游集散地和珍珠湾国际旅游目的地；打造百里黄金海岸旅游线、十万大山生态养生旅游线、中越跨境海陆旅游线三条旅游线路；加快白浪滩、西湾、金滩、红沙群岛四个景区建设，力争白浪滩创5A景区、西湾创4A景区。完善“吃住行游购娱”一体化服务设施，加快旅游码头、海鲜美食城、城市沙滩建设，尽快建成渭博·基斯顿、港宸国际、嘉威海港等高档滨海酒店，开发特色旅游纪念品。发展乡村旅游，完善簕山古渔村、竹山村等旅游示范村配套服务设施，建设一批农家乐。搭建旅游信息平台，推进智慧旅游建设。

繁荣商贸业。推进中心区集贸市场、桃花湾集贸市场、沙潭江汽贸园、金海海产品市场、中心区家居建材市场等大型专业市场建设，打造区域性消费集聚区，促进消费繁荣。推动沃尔玛、华联、南城百货等大型知名品牌进驻，鼓励连锁企业到社区、农村建立网点，推进乡镇农贸市场规范化建设。

发展房地产业。以中央商务区、桃花湾片区、常山新区为重点，以荣顾购物公园、阳光海岸等大型城市综合体为引领，完善城市生活设施配套，促进房地产健康发展。发展商业地产、旅游地产和工业地产，扩大房地产市场需求。打造一批高档商住小区，吸引高端企业和客户聚集。

壮大金融业。加强政银企协调沟通，拓宽融资渠道，为重点项目和企业发展提供金融支持和资金保障。加强资本市场建设，加大企业上市培育力度，推动符合条件的中小企业在全国股份转让系统挂牌。加快农村金融产品和服务方式创新，增强金融机构服务“三农”能力。实施“引金入港”工程，增加金融机构总量。加强对小额贷款公司和融资性担保行业的规范和监管，防范金融市场风险，促进金融有序发展。

六、加快港口交通设施建设，打造区域性国际枢纽

强化港口龙头地位，全力构建多层次、高效能、立体化的综合交通体系，做大做强港口物流业。

强化港口建设。以改造提升渔氵万港区、开发建设企沙港区为重点，加快港航设施建设。渔氵万港区加快513-516#泊位工程、信润石化配套码头、马鞍岭1-2#旅游码头工程建设，开工建设东湾第五作业区进港航道工程，推进501-502#泊位码头工程等项目前期工作，尽快启动30万吨泊位和航道建设。企沙港区加快钢铁基地专用码头建设，开工建设防城港电厂二期配套码头，推进金川配套码头、潭油作业区3-4#泊位等项目前期工作。加强茅岭、潭吉等中小港口港航设施建设。

加快交通建设。启动企沙至茅岭一级公路、马路至峒中公路改造建设，开展钦防高速公路改扩建、百色经崇左至防城港铁路前期工作。港口至东兴一级公路改造年内实现竣工。做好开工建设防城港通往河内高速公路和铁路的相关工作，尽快启动中越峒中—横模大桥建设。推进长途汽车客运站、公交车站、出租车站建设，加快建成城市交通综合枢纽。开展吴圩机场经上思至东兴试验区高速公路等项目的前期工作，推进防城港支线机场项目选址论证工作。

大力发展物流业。推进物流园区建设，抓好中国-东盟农产品（防城港）进出口物流中心、防城港国际汽车城、东盟农产品国际采购中心等项目建设。引进一批高质量的现代物流项目落户，吸引有实力的企业设立物流加工园，合资组建大型现代化物流公司，鼓励发展第三方、第四方物流，发展电子商务，打造辐射西南、中南地区和东盟国家的区域性物流中心。

优化交通服务环境。注重港铁联运，拓展“水转水”“水转铁”业务，强化全程综合物流配送服务，提高集疏运能力。推进集装箱多式联运，提高车船装卸效率。加快航贸服务中心建设，整合口岸通关，实行集中联检，打造“一站式通关”。完成防城港货场搬迁和运输信息指挥中心建设，推进散杂货作业生产管理系统建设，提高港口生产信息化水平。落实扶持港口发展政策，支持集装箱发展和航线拓展，吸引更多港航企业和中介企业入驻，努力打造航运枢纽。

七、强化“三农”工作，加快发展现代农业

加强农村基础设施建设，发展现代农业，构建新型农业经营体系，促进农民增收，激发农村经济社会活力。

加快农业产业化。实施农业产业化“336”工程，稳定粮食播种面积，培育发展农业龙头企业，建立优势农业产业基地。安排2000万元资金，重点扶持中药材、食用菌、坚果和“红金系列”产品等产业发展。发展林下种养，壮大林下经济。发展海水养殖和水产品加工，扩大畜牧业养殖规模。抓好菜篮子工程，建设200个蔬菜大棚。强化科技对农业支撑，引进农产品深加工项目，延伸农业发展产业链。发展农民专业合作社，新增农民合作组织40家。培育发展家庭农场等新型经营组织。

促进农民创业增收。深入实施农民收入倍增计划，打造“万元村屯”450个、“万元片区”22个、“示范农户”400个，以点带面推动全市农民增收。推进土地确权登记试点工作，探索农村土地流转、土地入股新模式。努力推动土地承包经营权向农业龙头企业、专业大户、家庭农场或农民合作社流转，发展多种形式适度规模经营。抓好扶贫工作，年内实现1.4万人脱贫。

强化农业基础设施建设。投资4亿元，抓好水利设施建设。加快大垌水库建设。完成灌区节水改造配套加固渠道160公里，改善灌溉面积8万亩。新增农村安全饮水人口3万人。实施村屯道路建设大会战，硬化通村、通屯道路300公里以上。

八、大力发展社会民生事业，努力构建和谐防城港

着力保障和改善民生，提高社会事业发展水平，为人民群众提供更多更好的公共服务，不断提高民生福祉，促进社会和谐。

提高社会保障水平。加快构建城乡一体化医疗保险体系，实现医疗保险制度从“3+1”模式向“2+1”模式转变。加强社会保险基金监管，推动社会保险机构整合，引导城乡居民参保续保。加强保障性住房建设和农村危房改造工作。积极扩大就业，新增城镇就业2.3万人。开展劳动力技能培训，促进农村劳动力转移就业。加大就业岗位开发，开展重点困难群体就业援助活动。扶持劳动者创业，引导和组织边民参与边境贸易3万人以上。创新人才工作机制，吸引更多国内外高层次人才落户防城港。

大力发展科技事业。加大科技投入，推进发明专利倍增计划，实施知识产权强县（市、区）工程。以高新科技产业园为载体，引进大企业、大集团到我市建立研发中心，吸引区内外知名科研机构落户我市。加快构建多元投入的科技孵化体系，年内新建科技企业孵化器2家。鼓励社会力量兴办各类科技中介服务机构，促进科技服务业发展。

优先发展教育事业。实施教育振兴行动计划和八大重点工程，开展“教育基础设施大提升”活动。抓好学前教育建设，新建5所乡镇幼儿园，在中心区新建一所自治区级示范性幼儿园。推动义务教育均衡发展，新建7所义务教育学校，加快市五中、六中等学校建设，逐步解决“大班额”问题。抓好市实验高中新校区建设和市高级中学扩建工程，打造一所高水平的自治区级示范高中。加快广西财经学院防城港校区二期建设，推进市理工学校新校区建设。争取引进一批名校名师和先进管理模式，不断提升教育水平。

推进文化体育事业发展。以边海文化特色为主线，探索市场化模式办好国际海上龙舟节、京族哈节等传统节庆，打造特色文化品牌。抓好《十万大山的女人》等影视文艺精品创作。加强与东盟各国的文化体育交流。推动群众文体活动常态化发展，丰富市民文化体育生活。推进“国门书店”建设工程。加强村级公共服务中心、社区文化活动中心、村村通广播电视等一批基层公共文化设施建设。抓好文化下乡，实施农村社区公益电影放映工程。

发展医疗卫生事业。全面推进创建自治区卫生城市工作。加快完善城市医院、社区卫生服务中心布局，建设中心区综合医院，加快金海湾、桃花湾社区卫生服务中心建设。投入700万元，为全市280多个村级卫生室配备医疗设备，提高基层医疗卫生服务水平。完善新型农村合作医疗制度，抓好新农合“一卡通”试点。推进公立医院改革。实施防治艾滋病攻坚工程、母婴健康“一免二补”幸福工程。落实计划生育政策，推进诚信计生。

强化社会治理。加强服务创新，提高社会治理水平，有效预防和化解社会矛盾。推进平安法治防城港建设，加强社会治安综合立体化治理，严厉打击重特大刑事犯罪活动，不断提高人民群众的安全感和满意度。开展安全生产隐患排查整治，加强食品药品安全监管，遏制重特大安全事故。加强国防建设，强化国家安全工作，加强边海防联防联控，保持打击走私的高压态势。推进社会治理信息化建设，提高引导和应对网络舆情能力。创新信访工作机制，维护群众合法权益。

加强生态文明建设。开展生态环境综合整治，落实主体功能区制度，加强红树林、十万大山、防城江等自然保护区及流域保护，强化水源地保护。深入开展环境安全隐患大排查大整治，加强港口粉尘治理，建成市区PM2.5自动监测系统。加快推进城镇污水、垃圾处理设施及配套建设。深入开展“美丽家园·清洁乡村”活动，多渠道筹措和整合资金，健全常态化机制。加强垃圾分类处理，对生活垃圾实行减量化、资源化。加强农村环境综合整治，实施村屯绿化美化工程，条件好的村屯要率先进行“穿衣戴帽”，打造一批“美丽家园”示范村。

实施为民办实事工程。筹措9.25亿元，实施城建、交通、水利、文化、卫生、安居、教育、生态、社保、平安等十大惠民工程建设。

九、加强政府自身建设，不断提高行政效能

深化行政体制改革，创新行政管理方式，增强政府公信力和执行力，全面正确履行政府职能，提高科学管理水平，打造法治政府、服务型政府和廉洁政府。

深化审批制度改革。深化重点领域和关键环节改革，推动改革创新取得新的突破。深化行政审批制度改革，简化审批流程，公开审批条件，减少审批数量，提高监管力度。抓好行政审批事项“接、放、管”工作。加快推进财税、金融、投资、价格等重点领域改革。探索试行负面清单管理，逐步建立新的投资准入管理体制。

提高法治工作水平。加强法制建设，严格规范行政执法。推进政务公开，完善政务服务中心综合服务功能，提高行政透明度，让权力在阳光下运行。加强行政监察和审计监督。自觉接受人大和政协的监督，认真办理人大代表建议、议案和政协委员提案。认真听取各民主党派、工商联、无党派人士、人民团体和社会各界人士的意见。

加强政府执行力。深入开展党的群众路线教育实践活动，以“七个提升”工作为抓手，全面提高政府执行力，切实解决群众反映的突出问题。以踏石留印、抓铁有痕的魄力，全力推动经济快速发展和社会各项事业全面进步，做到工作不落实不放过，项目不推进不放过，局面打不开不放过，民生不改善不放过。强化行政问责，促进公务人员履职到位、尽责到位。

加强作风和廉政建设。认真落实中央“八项规定”和国务院“约法三章”的精神，加强自身建设，改进工作作风。精文简会，切实改进文风会风。规范公务用车管理，严格执行公务接待制度和标准，规范因公出国（境）管理。推进财政预决算公开。落实党风廉政建设责任制，严格遵守廉洁从政各项规定，营造风清气正的政务环境。

各位代表！骏马奔驰千里路，港城腾跃万众心。我市正站在新的历史起点上，发展任重而道远，让我们紧密团结在以习近平同志为总书记的党中央周围，在市委的坚强领导下，同心同德，克难攻坚，改革创新，奋发图强，为加快实现“在全区率先发展、在沿海后来居上、迈入沿海开放城市第一集团”而努力奋斗！
